# Supplementary material for: The Different Effects of Organic Amines on Synthetic Metal Phosphites/Phosphates
Source: Materials (Basel). 2020 Apr 9;13(7):1752. doi: 10.3390/ma13071752 (PMC7178704; doi:10.3390/ma13071752)
Supplement: Supplementary file 1 [file materials-13-01752-s001.pdf]

Supporting Information

The different effects of organic amines on synthetic metal phosphites/phosphates

Xuelei Wang [1,3,*], Zhaojun Dong [2], Chao Meng [1], Wei Wang [1], Hairui Yang [1], Xizhun, Zhuo [3] and Shaobin Yang [1,3*]

[a]College of Materials Science and Engineering, Liaoning Technical University, Fuxin, Liaoning 123000, P. R. China

[b]College of New Energy and Environment, Jilin University, Changchun, Jilin 130012, P. R. China

[c]College of Mining, Liaoning Technical University, Fuxin, Liaoning 123000, P. R. China

Table S1a Bond lengths [Å] and bond angles [°] for compound **1**

P(1)-O(2)	1.504(3)	O(2)-P(1)-O(4)	110.26(17)
P(1)-O(3)	1.508(3)	O(3)-P(1)-O(4)	106.89(17)
P(1)-O(1)	1.535(3)	O(1)-P(1)-O(4)	107.88(18)
P(1)-O(4)	1.540(3)	O(1)-Al(1)-O(1)#1	113.5(2)
Al(1)-O(1)	1.717(3)	O(1)-Al(1)-O(4)#2	109.19(15)
Al(1)-O(1)#1	1.718(3)	O(1)#1-Al(1)-O(4)#2	106.07(16)
Al(1)-O(4)#2	1.722(3)	O(1)-Al(1)-O(4)#3	106.07(16)
Al(1)-O(4)#3	1.722(3)	O(1)#1-Al(1)-O(4)#3	109.19(15)
O(4)-Al(1)#4	1.722(3)	O(4)#2-Al(1)-O(4)#3	113.0(2)
N(1)-C(1)	1.493(5)	P(1)-O(1)-Al(1)	156.6(2)
C(1)-C(1)#5	1.550(8)	P(1)-O(4)-Al(1)#4	150.7(2)
C(1)-C(2)	1.584(8)	N(1)-C(1)-C(1)#5	109.4(4)
O(2)-P(1)-O(3)	113.16(18)	N(1)-C(1)-C(2)	101.0(4)
O(2)-P(1)-O(1)	108.15(17)	C(1)#5-C(1)-C(2)	117.1(5)
O(3)-P(1)-O(1)	110.37(18)		

Symmetry transformations used to generate equivalent atoms:#1 -x+3/2,-y+3/2,z; #2 -x+3/2,y,z+1/2; #3 x,-y+3/2,z+1/2; #4 -x+3/2,y,z-1/2;#5 -x,-y+2,-z+1.

Table S1b Bond lengths [Å] and bond angles [°] for compound **2**

Ga(1)-O(5)	1.941(4)	O(5)-H(5O)	0.9863
Ga(1)-O(5)#1	1.951(4)	N(1)-C(1)	1.481(8)
Ga(1)-O(2)#1	1.951(4)	N(1)-H(1A)	0.8900
Ga(1)-O(1)	1.956(5)	N(1)-H(1B)	0.8899
Ga(1)-O(3)	1.998(4)	N(1)-H(1C)	0.8900
Ga(1)-O(4)	1.998(4)	N(2)-C(2)	1.502(10)
P(1)-O(6)	1.502(5)	N(2)-H(2A)	0.8900
P(1)-O(2)	1.511(5)	N(2)-H(2B)	0.8901
P(1)-O(1)	1.521(5)	N(2)-H(2C)	0.8900
P(1)-H(1P)	1.3333	C(1)-C(2)	1.520(9)
P(2)-O(7)	1.497(5)	C(1)-H(1D)	0.9700
P(2)-O(3)	1.524(5)	C(1)-H(1E)	0.9700
P(2)-O(4)#2	1.524(5)	C(2)-C(3)	1.486(8)
P(2)-H(2P)	1.3721	C(2)-H(2)	0.9800
O(2)-Ga(1)#2	1.951(4)	C(3)-H(3A)	0.9600
O(4)-P(2)#1	1.524(5)	C(3)-H(3B)	0.9600
O(5)-Ga(1)#2	1.951(4)	C(3)-H(3C)	0.9600

O(5)-Ga(1)-O(5)#1	177.97(6)	Ga(1)-O(5)-H(5O)	114.9
O(5)-Ga(1)-O(2)#1	89.10(19)	Ga(1)#2-O(5)-H(5O)	112.8
O(5)#1-Ga(1)-O(2)#1	92.89(19)	C(1)-N(1)-H(1A)	109.6
O(5)-Ga(1)-O(1)	91.09(19)	C(1)-N(1)-H(1B)	109.1
O(5)#1-Ga(1)-O(1)	86.92(19)	H(1A)-N(1)-H(1B)	109.5
O(2)#1-Ga(1)-O(1)	179.62(19)	C(1)-N(1)-H(1C)	109.7
O(5)-Ga(1)-O(3)	91.90(18)	H(1A)-N(1)-H(1C)	109.5
O(5)#1-Ga(1)-O(3)	88.53(18)	H(1B)-N(1)-H(1C)	109.5
O(2)#1-Ga(1)-O(3)	89.2(2)	C(2)-N(2)-H(2A)	109.4
O(1)-Ga(1)-O(3)	90.4(2)	C(2)-N(2)-H(2B)	109.5
O(5)-Ga(1)-O(4)	87.02(17)	H(2A)-N(2)-H(2B)	109.5
O(5)#1-Ga(1)-O(4)	92.57(17)	C(2)-N(2)-H(2C)	109.6
O(2)#1-Ga(1)-O(4)	90.5(2)	H(2A)-N(2)-H(2C)	109.5
O(1)-Ga(1)-O(4)	89.9(2)	H(2B)-N(2)-H(2C)	109.5
O(3)-Ga(1)-O(4)	178.88(18)	N(1)-C(1)-C(2)	113.6(5)
O(6)-P(1)-O(2)	112.6(3)	N(1)-C(1)-H(1D)	108.8
O(6)-P(1)-O(1)	110.7(3)	C(2)-C(1)-H(1D)	108.8
O(2)-P(1)-O(1)	112.4(3)	N(1)-C(1)-H(1E)	108.8
O(6)-P(1)-H(1P)	107.4	C(2)-C(1)-H(1E)	108.8
O(2)-P(1)-H(1P)	106.4	H(1D)-C(1)-H(1E)	107.7
O(1)-P(1)-H(1P)	107.0	C(3)-C(2)-N(2)	110.1(5)
O(7)-P(2)-O(3)	111.4(3)	C(3)-C(2)-C(1)	107.9(5)
O(7)-P(2)-O(4)#2	111.4(3)	N(2)-C(2)-C(1)	113.3(6)
O(3)-P(2)-O(4)#2	114.0(3)	C(3)-C(2)-H(2)	108.5
O(7)-P(2)-H(2P)	124.2	N(2)-C(2)-H(2)	108.5
O(3)-P(2)-H(2P)	97.6	C(1)-C(2)-H(2)	108.5
O(4)#2-P(2)-H(2P)	97.3	C(2)-C(3)-H(3A)	109.5
P(1)-O(1)-Ga(1)	132.9(3)	C(2)-C(3)-H(3B)	109.5
P(1)-O(2)-Ga(1)#2	130.9(3)	H(3A)-C(3)-H(3B)	109.5
P(2)-O(3)-Ga(1)	133.5(3)	C(2)-C(3)-H(3C)	109.5
P(2)#1-O(4)-Ga(1)	132.4(3)	H(3A)-C(3)-H(3C)	109.5
Ga(1)-O(5)-Ga(1)#2	131.4(2)	H(3B)-C(3)-H(3C)	109.5

Symmetry transformations used to generate equivalent atoms: #1 -x+1,y-1/2,-z+1/2; #2 -x+1,y+1/2,-z+1/2.

Table S1c Bond lengths [Å] and bond angles [°] for compound **3**

In(1)-O(7)	2.072(3)	O(3)-In(1)-In(2)#2	36.23(8)
In(1)-O(6)	2.089(3)	O(9)#3-In(2)-O(1)	97.36(13)
In(1)-O(8)	2.096(3)	O(9)#3-In(2)-O(2)#4	92.61(12)
In(1)-O(5)#1	2.143(3)	O(1)-In(2)-O(2)#4	94.61(13)
In(1)-O(4)	2.208(3)	O(9)#3-In(2)-O(10)	170.72(13)
In(1)-O(3)	2.218(3)	O(1)-In(2)-O(10)	89.71(13)
In(1)-In(2)#2	3.5119(4)	O(2)#4-In(2)-O(10)	92.80(14)
In(2)-O(9)#3	2.069(3)	O(9)#3-In(2)-O(3)#5	91.69(13)
In(2)-O(1)	2.082(3)	O(1)-In(2)-O(3)#5	98.15(12)
In(2)-O(2)#4	2.114(3)	O(2)#4-In(2)-O(3)#5	165.90(12)
In(2)-O(10)	2.148(3)	O(10)-In(2)-O(3)#5	81.33(13)
In(2)-O(3)#5	2.165(3)	O(9)#3-In(2)-O(4)#5	88.92(14)
In(2)-O(4)#5	2.237(3)	O(1)-In(2)-O(4)#5	170.71(16)
In(2)-In(1)#5	3.5118(4)	O(2)#4-In(2)-O(4)#5	91.92(12)
P(1)-O(1)	1.505(3)	O(10)-In(2)-O(4)#5	83.37(13)
P(1)-O(5)	1.511(3)	O(3)#5-In(2)-O(4)#5	74.74(11)
P(1)-O(4)	1.548(3)	O(9)#3-In(2)-In(1)#5	91.93(9)
P(1)-H(1P)	1.3205	O(1)-In(2)-In(1)#5	134.86(9)
P(2)-O(6)	1.513(4)	O(2)#4-In(2)-In(1)#5	129.10(9)
P(2)-O(2)	1.523(3)	O(10)-In(2)-In(1)#5	78.82(9)
P(2)-O(8)#3	1.526(3)	O(3)#5-In(2)-In(1)#5	37.26(8)
P(2)-H(2P)	1.2239	O(4)#5-In(2)-In(1)#5	37.53(8)
P(3)-O(9)	1.507(4)	O(1)-P(1)-O(5)	115.7(2)
P(3)-O(7)#2	1.509(3)	O(1)-P(1)-O(4)	110.5(2)
P(3)-O(3)	1.558(3)	O(5)-P(1)-O(4)	111.53(19)
P(3)-H(3P)	1.3038	O(1)-P(1)-H(1P)	104.6
O(2)-In(2)#6	2.114(3)	O(5)-P(1)-H(1P)	109.8
O(3)-In(2)#2	2.165(3)	O(4)-P(1)-H(1P)	103.7
O(4)-In(2)#2	2.237(3)	O(6)-P(2)-O(2)	111.60(19)
O(5)-In(1)#3	2.143(3)	O(6)-P(2)-O(8)#3	114.90(18)
O(7)-P(3)#5	1.509(3)	O(2)-P(2)-O(8)#3	107.52(19)
O(8)-P(2)#1	1.526(3)	O(6)-P(2)-H(2P)	102.6
O(9)-In(2)#1	2.069(3)	O(2)-P(2)-H(2P)	105.3
O(10)-H(1)	0.8322	O(8)#3-P(2)-H(2P)	114.6
O(10)-H(2)	0.7933	O(9)-P(3)-O(7)#2	113.8(2)
		O(9)-P(3)-O(3)	109.1(2)
O(7)-In(1)-O(6)	88.47(14)	O(7)#2-P(3)-O(3)	110.26(19)
O(7)-In(1)-O(8)	102.07(14)	O(9)-P(3)-H(3P)	108.2
O(6)-In(1)-O(8)	169.43(14)	O(7)#2-P(3)-H(3P)	109.5
O(7)-In(1)-O(5)#1	98.20(13)	O(3)-P(3)-H(3P)	105.7
O(6)-In(1)-O(5)#1	84.64(11)	P(1)-O(1)-In(2)	140.3(2)
O(8)-In(1)-O(5)#1	93.01(14)	P(2)-O(2)-In(2)#6	130.30(19)

O(7)-In(1)-O(4)	93.79(12)	P(3)-O(3)-In(2)#2	130.05(19)
O(6)-In(1)-O(4)	93.20(15)	P(3)-O(3)-In(1)	122.83(17)
O(8)-In(1)-O(4)	86.92(11)	In(2)#2-O(3)-In(1)	106.51(13)
O(5)#1-In(1)-O(4)	167.74(11)	P(1)-O(4)-In(1)	130.21(17)
O(7)-In(1)-O(3)	164.97(14)	P(1)-O(4)-In(2)#2	125.01(17)
O(6)-In(1)-O(3)	83.22(14)	In(1)-O(4)-In(2)#2	104.37(12)
O(8)-In(1)-O(3)	86.65(14)	P(1)-O(5)-In(1)#3	145.5(2)
O(5)#1-In(1)-O(3)	93.48(12)	P(2)-O(6)-In(1)	144.4(2)
O(4)-In(1)-O(3)	74.27(10)	P(3)#5-O(7)-In(1)	142.7(2)
O(7)-In(1)-In(2)#2	131.56(9)	P(2)#1-O(8)-In(1)	143.7(2)
O(6)-In(1)-In(2)#2	89.14(10)	P(3)-O(9)-In(2)#1	132.6(2)
O(8)-In(1)-In(2)#2	84.43(10)	In(2)-O(10)-H(1)	130.2
O(5)#1-In(1)-In(2)#2	129.68(9)	In(2)-O(10)-H(2)	123.2
O(4)-In(1)-In(2)#2	38.10(7)	H(1)-O(10)-H(2)	106.4

Symmetry transformations used to generate equivalent atoms: #1 -x-2,y-1/2,-z-2; #2 x-1,y,z; #3 -x-2,y+1/2,-z-2; #4 x+1,y,z+1; #5 x+1,y,z; #6 x-1,y,z-1.

Table S1d Bond lengths [Å] and bond angles [°] for compound **4**

In(1)-O(4)	2.109(3)	O(8)-In(2)-O(7)	173.30(14)
In(1)-O(1)	2.111(3)	O(8)-In(2)-O(9)	88.15(14)
In(1)-O(3)	2.114(4)	O(7)-In(2)-O(9)	88.20(14)
In(1)-O(2)	2.119(4)	O(8)-In(2)-O(10)	91.39(14)
In(1)-O(11)	2.129(4)	O(7)-In(2)-O(10)	83.61(13)
In(1)-O(6)	2.175(3)	O(9)-In(2)-O(10)	98.72(14)
In(2)-O(8)	2.084(3)	O(8)-In(2)-O(12)	91.50(14)
In(2)-O(7)	2.104(3)	O(7)-In(2)-O(12)	92.75(14)
In(2)-O(9)	2.108(3)	O(9)-In(2)-O(12)	173.59(15)
In(2)-O(10)	2.117(3)	O(10)-In(2)-O(12)	87.69(15)
In(2)-O(12)	2.190(4)	O(8)-In(2)-O(5)	87.98(14)
In(2)-O(5)	2.203(3)	O(7)-In(2)-O(5)	97.26(13)
P(1)-O(1)	1.515(4)	O(9)-In(2)-O(5)	84.42(15)
P(1)-O(10)	1.527(3)	O(10)-In(2)-O(5)	176.77(16)
P(1)-O(11)#1	1.532(4)	O(12)-In(2)-O(5)	89.17(17)

P(1)-H(1P)	1.3711	O(1)-P(1)-O(10)	113.8(2)
P(2)-O(4)	1.509(3)	O(1)-P(1)-O(11)#1	112.4(2)
P(2)-O(9)	1.515(3)	O(10)-P(1)-O(11)#1	110.8(2)
P(2)-O(7)#2	1.529(4)	O(1)-P(1)-H(1P)	102.3
P(2)-H(2P)	1.3383	O(10)-P(1)-H(1P)	106.8
P(3)-O(8)#3	1.504(3)	O(11)#1-P(1)-H(1P)	110.1
P(3)-O(3)#4	1.510(4)	O(4)-P(2)-O(9)	112.4(2)
P(3)-O(2)	1.515(4)	O(4)-P(2)-O(7)#2	110.3(2)
P(3)-H(3P)	1.3340	O(9)-P(2)-O(7)#2	110.2(2)
O(3)-P(3)#1	1.510(4)	O(4)-P(2)-H(2P)	106.0
O(6)-H(6A)	0.7504	O(9)-P(2)-H(2P)	111.2
O(6)-H(6B)	0.7965	O(7)#2-P(2)-H(2P)	106.5
O(7)-P(2)#5	1.529(4)	O(8)#3-P(3)-O(3)#4	111.8(2)
O(8)-P(3)#6	1.504(3)	O(8)#3-P(3)-O(2)	110.2(2)
O(11)-P(1)#4	1.532(3)	O(3)#4-P(3)-O(2)	112.4(2)
O(12)-H(12A)	0.8167	O(8)#3-P(3)-H(3P)	107.0
O(12)-H(12B)	0.8389	O(3)#4-P(3)-H(3P)	106.4
O(1W)-H(1W)	0.9380	O(2)-P(3)-H(3P)	108.7
O(1W)-H(2W)	0.9647	P(1)-O(1)-In(1)	129.4(2)
O(4)-In(1)-O(1)	96.64(13)	P(3)-O(2)-In(1)	138.6(2)
O(4)-In(1)-O(3)	85.60(14)	P(3)#1-O(3)-In(1)	139.7(2)
O(1)-In(1)-O(3)	89.77(14)	P(2)-O(4)-In(1)	133.83(19)
O(4)-In(1)-O(2)	91.14(15)	In(1)-O(6)-H(6A)	125.1
O(1)-In(1)-O(2)	83.06(12)	In(1)-O(6)-H(6B)	135.9
O(3)-In(1)-O(2)	171.74(15)	H(6A)-O(6)-H(6B)	96.5
O(4)-In(1)-O(11)	85.57(13)	P(2)#5-O(7)-In(2)	128.1(2)
O(1)-In(1)-O(11)	177.23(14)	P(3)#6-O(8)-In(2)	140.5(2)
O(3)-In(1)-O(11)	92.07(14)	P(2)-O(9)-In(2)	130.8(2)
O(2)-In(1)-O(11)	95.25(16)	P(1)-O(10)-In(2)	137.9(2)
O(4)-In(1)-O(6)	173.98(15)	P(1)#4-O(11)-In(1)	138.4(2)
O(1)-In(1)-O(6)	89.36(14)	In(2)-O(12)-H(12A)	135.1
O(3)-In(1)-O(6)	94.85(16)	In(2)-O(12)-H(12B)	129.5
O(2)-In(1)-O(6)	89.19(17)	H(12A)-O(12)-H(12B)	92.1
O(11)-In(1)-O(6)	88.42(14)	H(1W)-O(1W)-H(2W)	88.7

Symmetry transformations used to generate equivalent atoms: #1 -x+1,y+1/2,-z+1; #2 -x,y-1/2,-z; #3h n -x,y-1/2,-z+1; #4 -x+1,y-1/2,-z+1; #5 -x,y+1/2,-z; #6 -x,y+1/2,-z+1.
